# Supplementary material for: Prevalence and risk factors of scabies among orphans: A cross-sectional study in Bangladesh
Source: PLoS Negl Trop Dis. 2025 Oct 29;19(10):e0013671. doi: 10.1371/journal.pntd.0013671 (PMC12591429; doi:10.1371/journal.pntd.0013671)
Supplement: S1 File — (DOCX) [file pntd.0013671.s001.docx]

**Title: Prevalence and risk factors of scabies among children living in orphanages of Bangladesh: a cross-sectional study**

**Data Collection Form**

[Please read the following questions carefully. Encircle the code according to your response.]

**ID. No: …………. Date: ……………….**

1. **Sociodemographic characteristics**

| **No** | **Questions** | **Responses** | **Code** |
| --- | --- | --- | --- |
| **1.1** | Gender | Male | 1 |
|  |  | Female | 2 |
| **1.2** | Age | …………… years |  |
| **1.3** | Number of children in the orphanage |  | |
| **1.4** | Number of students using the same toilet |  | |
| **1.5** | Number of students per dormitory/room |  | |
| **1.6** | Sleeping place | On bed | 1 |
|  |  | On floor | 2 |
| **1.7** | Number of baths | At least once daily | 1 |
|  |  | Less than once daily | 2 |
| **1.8** | Sleeping with others | Yes | 1 |
|  |  | No | 2 |
| **1.9** | Sharing of beddings, clothes or toilet stuffs | Yes | 1 |
|  |  | No | 2 |
| **1.10** | Usage of soap for baths | Yes | 1 |
|  |  | No | 2 |
| **1.11** | Finger nails always cut short | Yes | 1 |
|  |  | No | 2 |
| **1.12** | Ironing of clothes and bedding | Yes | 1 |
|  |  | No | 2 |
| **1.13** | Pruritus in the close entourage | Yes | 1 |
|  |  | No | 2 |
| **1.14** | Complaining of pruritus | Yes | 1 |
|  |  | No | 2 |

1. **Scabies related questions**

| **No** | **Questions** | **Responses** | **Code** |
| --- | --- | --- | --- |
| **1.15** | **Itching/lesion present at** |  |  |
|  | interdigital spaces | Yes/No/ Not examined |  |
|  | hand | Yes/No/ Not examined |  |
|  | wrist | Yes/No/ Not examined |  |
|  | arm | Yes/No/ Not examined |  |
|  | elbow | Yes/No/ Not examined |  |
|  | axilla | Yes/No/ Not examined |  |
|  | leg | Yes/No/ Not examined |  |
|  | foot | Yes/No/ Not examined |  |
|  | abdomen | Yes/No/ Not examined |  |
|  | thorax | Yes/No/ Not examined |  |
|  | mamilla/peri-mamillar area | Yes/No/ Not examined |  |
|  | back | Yes/No/ Not examined |  |
|  | buttock | Yes/No/ Not examined |  |
|  | genital/inguinal area | Yes/No/ Not examined |  |
|  | head (scalp/ neck/face) | Yes/No/ Not examined |  |
| **1.16 Confirmed scabies meets at least one of the following criteria:** | | | |
| A1 | mites, eggs or feces on light microscopy of skin samples | Yes | 1 |
|  |  | No | 2 |
| A2 | mites, eggs or feces visualized on an individual using a high-powered imaging device | Yes | 1 |
|  |  | No | 2 |
| A3 | mite visualized on an individual using dermoscopy | Yes | 1 |
|  |  | No | 2 |
| **B** | **Clinical scabies meets at least one of the following criteria:** |  |  |
| B1 | scabies burrows | Yes | 1 |
|  |  | No | 2 |
| B2 | typical lesions affecting male genitalia | Yes | 1 |
|  |  | No | 2 |
| B3 | typical lesions in a typical distribution and two history features | Yes | 1 |
|  |  | No | 2 |
| **C** | **Suspected scabies meets one of the following criteria:** |  |  |
| C1 | typical lesions in a typical distribution and one history feature | Yes | 1 |
|  |  | No | 2 |
| C2 | atypical lesions or atypical distribution and two history features | Yes | 1 |
|  |  | No | 2 |
| **1.17** | **Severity assessment:** |  |  |
|  | **Mild (1–10 lesions)** | Yes | 1 |
|  |  | No | 2 |
|  | **Moderate (11-49 lesions)** | Yes | 1 |
|  |  | No | 2 |
|  | **Severe (≥50 lesions)** | Yes | 1 |
|  |  | No | 2 |
| **1.18** | **Other skin disease** | Yes | 1 |
|  |  | No | 2 |
| **1.19** | **If yes in 1.18, what are the provisional diagnosis** |  | |

**Thank you for your participation.**
