# Supplementary material for: Prevalence and risk factors of scabies among orphans: A cross-sectional study in Bangladesh
Source: PLoS Negl Trop Dis. 2025 Oct 29;19(10):e0013671. doi: 10.1371/journal.pntd.0013671 (PMC12591429; doi:10.1371/journal.pntd.0013671)
Supplement: Attachment — Submitted filename: 5000136. Review note V 2-JH.docx [file pntd.0013671.s004.docx]

29 March 2025

Re: Resubmission of manuscript *"Prevalence and risk factors of scabies among orphans: a cross-sectional study in Bangladesh" (PNTD-D-24-01624)*

To

Editor-in-Chief,

PLOS Neglected Tropical Diseases

Dear Editor-in-Chief,

Thank you for the thorough review of our manuscript, *"Prevalence and risk factors of scabies among orphans: a cross-sectional study in Bangladesh" (PNTD-D-24-01624).* We appreciate the careful review and constructive suggestions to improve the manuscript. We have addressed the comments of the editor and the reviewer(s) and amended the manuscript accordingly. The revision has been developed in consultation with all coauthors, and each author has given approval to the final form of this revision.

Thank you for your consideration.

Sincerely,

**Mohammad Jahid Hasan**

Tropical Disease and Health Research Center

Dhaka, Bangladesh-1211

Email: [dr.jahid61@gmail.com](mailto:dr.jahid61@gmail.com)

Cell: 01757818973

**Point-by-point response**

**PNTD-D-24-01624
Prevalence and risk factors of scabies among orphans: a cross-sectional study in Bangladesh**

**Copyrights issue:**

The clip-art used in the current version of Figure 1 was sourced from [Wikimedia Commons](https://commons.wikimedia.org/wiki/File:Human_body_with_blank_labels.svg#Licensing). This image is made available under the [Creative Commons CC0 1.0 Universal Public Domain Dedication](https://creativecommons.org/publicdomain/zero/1.0/), through which the creator has waived all rights to the work worldwide under copyright law, including related and neighboring rights, to the extent permitted by law. Consequently, the image may be freely used, modified, and distributed for any purpose, including commercial applications, without requiring permission.

**Additional Editor Comments:**

We have now, after some delay, received all reviewer comments. Whilst they are generally positive, as you can see from reading them our reviewers still considered your manuscript needs further work. In addition to the comments provided below, I would also like to flag up that whilst the manuscript is generally well written, its grammar and word use could be improved. One reviewer specifically asked me to suggest to you that you should pay further attention to this, so as to improve the manuscript for the reader.

Note, one of the reviewers (reviewer 1) is a medical statistician (with a background in scabies work), please follow their guidance in your revision of the manuscript, and if you think it contradicts anything said by one of the other reviewers please follow reviewers 1’s instructions which take primacy.

In addition to the reviewer comments, there are a few issues which I as editor would like you to address. I outline these below.

***Authors’ response:*** *Thank you for providing a detailed review of our manuscript. We appreciate the constructive suggestions and have carefully revised the manuscript according to the recommendations, ensuring that all concerns are addressed. We sincerely appreciate the time and effort invested in reviewing our work and believe these revisions have strengthened the manuscript.*

**RE: SARCOPTES SCABIEI VAR. HOMINIS**
Line 44: ‘human parasite mite Sarcoptes scabiei var. hominis.’
Change text to: ‘parasitic mite Sarcoptes scabiei’. The var. hominis and reference to ‘human’ is outdated and should be removed. We have known for a decade that the phylogenetics of S. scabiei do not support either (1) a uniform host-specific set of variants (as indicated in your manuscript) or (2) panmixia, in which all S. scabiei clades – across host species – interbreed. Make this change, and if interested, see the following paper (of which I am not an author or otherwise involved): Andriantsoanirina V, Ariey F, Izri A, Bernigaud C, Fang F, Charrel R, Foulet F, Botterel F, Guillot J, Chosidow O, Durand R. Sarcoptes scabiei mites in humans are distributed into three genetically distinct clades. Clin Microbiol Infect. 2015 Dec;21(12):1107-14. doi: 10.1016/j.cmi.2015.08.002.

***Authors’ response:*** *Thank you for your insightful comment and for providing relevant literature on the genetic diversity of Sarcoptes scabiei. We have revised the text accordingly, replacing "human parasite mite Sarcoptes scabiei var. hominis" with "parasitic mite Sarcoptes scabiei", as suggested (page 3, line 44).*

**RE: PARTIAL EXAMINATIONS AND REPORTING OF SCABIES SIGN BODY DISTRIBUTION**
In pages 115-116 you write: “examination of sensitive areas such as the groin, buttocks, breasts and torso were skipped as it was not practical in the field setting.”
Please provide a justification for why this was not considered practical in a field setting. Many epidemiological studies on scabies, including ones I've been involved with personally in both low and high income settings, have carried out this in the field. Notably you use IACS criteria for clinical scabies which requires at least one of the following three to be present: “B1: scabies burrows; B2: Typical lesions affecting male genitalia; B3: Typical lesions in a typical distribution and two history features.” If you didn't examine male genitalia, how can you say lesions there are typical or not (and as I outline in a minute, you do report that many of your study participants had scabies signs there). In lines 153-154 you write: ‘Genital and inguinal area was the most commonly reported site of scabies lesion found in almost 65% of the infected children followed by interdigital space, hands, legs and abdomen’. This is also shown in figure 1. Given you state that you didn't in fact examine genitalia, but genitalia was the most commonly reported site, explain this contradiction. Was this based on patient/other self report? If so do your records disaggregate distribution of scabies signs between confirmed signs by examination and declared signs by patient/other report? If it does, please rectify (and disaggregate) your text and Figure 1 so this is clear to the reader. If it doesn't, provide justification for how your reported scabies sign distribution statistics can be reliably interpreted.

***Authors’ response:*** *Thank you for your detailed comment and for highlighting the need for clarification regarding the examination of sensitive areas. We have now clearly specified the approach taken in the field setting. In fact, participants who exhibited clinical manifestations of scabies and reported itching in private areas were asked whether they had similar lesions in these regions. If they confirmed the presence of lesions, a physical examination was conducted in a private setting with appropriate privacy measures. Additionally, a female physician was present for female children and male children under the age of 12, while a male physician was present for older male children. This clarification has been incorporated into Page 5, lines 123-133 to ensure better understanding and clarity of the examination process.*

**RE: DATA COLLECTION MATERIALS**
Include in supplementary material an English translation of the questionnaire, and any other data collection forms used (for examination etc.), so readers can know what was actually asked.

***Authors’ response:*** *We have included the English translation of the questionnaire as Supplementary File 1 to provide readers with a clear understanding of the study materials.*

**RE HYGIENE**
Throughout the manuscript there are a number of mentions of personal hygiene. In most cases you do not define exactly what you mean and imply a relationship to scabies prevalence or risk which was not found in your own study (despite looking for it), and is not supported by the existing balance of the overall evidence base or the International Alliance for the Control of Scabies. For example, on line 212-215 you write: “Personal hygiene is one of the major influencing factors of scabies among children. It is evidenced that children who maintain less personal hygiene, and use shared personal stuffs have a higher odd of having scabies [29]”. Similarly, on line 224-226 you state: “Though the significance of personal hygiene in scabies incidence is widely acknowledged [29], our study did not distinctly establish a conclusive association with hygiene practices.” In fact, the reference you give to support these two statements ( [29] Engelman et al. 2019. Lancet) says the opposite of what you claim re personal hygiene. Its only discussion of hygiene is as follows: “Scabies is often incorrectly attributed to poor hygiene, which can lead to stigma, shame, and reduced health seeking. However, hygiene and handwashing do not affect the mite or transmission,95 and highly effective control has been shown without any measures addressing hygiene or environment (see section on population-level control),96 suggesting that associations with situations of poverty and disadvantage are probably due to poor access to health care and treatment, or the effects of overcrowding.21,97”

***Authors’ reply:*** *We sincerely appreciate your thoughtful and detailed feedback on this issue. You have raised an important point regarding the role of personal hygiene in scabies transmission, and we acknowledge the need for greater clarity in our discussion. While some studies, such as Gupta et al. (DOI: 10.1111/tmi.14058), have suggested a potential link between certain personal behaviors (e.g., infrequent bathing and lack of soap use) and scabies, as mentioned in the present version of our discussion, the broader literature, including Engelman et al. (2019), does not support a strong association. Moreover, our own study, including the present one and the previous one, did not find a significant relationship between personal hygiene practices and scabies prevalence.*

*In response to your comments, we have carefully revised our manuscript to ensure that our discussion accurately reflects the current evidence base. We have also clarified any statements that may have previously implied a direct association between personal hygiene and scabies risk. We truly appreciate your insights, which have helped us refine our interpretation and improve the clarity of our work.*

*****Please make the following changes in your updated manuscript, or provide a strong point-by-point defence for why not.*****

Line 59: Remove “hygiene conditions”. No reference is provided for this statement.

***Authors reply:*** *Thank you for your suggestion. We have removed "hygiene conditions" from this section and revised as:*

*“Overcrowded living conditions, malnutrition, shared sleeping arrangements and sharing of clothes and towels with those who are already infected are common risk factors for this disease.” (Page 3, Line 57-59)*

Line 59-61: You write: “Overcrowded living conditions, shared sleeping arrangements, sharing of clothes and towels, poor hygiene, malnutrition, and travel to scabies outbreak areas are common risk factors for scabies [9, 10].” Remove “poor hygiene” from this list. The results of the study you give as reference 9 (Melese et al. 2023. BMC Trop Med Health) does support your inclusion of clothes sharing (at least re sharing with a scabies case), hence I am not saying to remove that, but they do not report results showing ‘personal hygiene’ is a risk factor. Your reference10 (Azene et al 2020. BMC Infect Dis) does not support your statement, in fact the opposite. Its results state: (A) “Association between frequency of bath and scabies infestation… the random effect model of a pooled odds ratio of frequency of the bath were not statically significant (OR: 1.47, 95% CI: 0.59, 3.64)”. (B) “Association of washing hand with soap and scabies infestation… The overall pooled odds ratio result of this study revealed that washing hands with soap had no statistically significant association with scabies infestation (OR: 0.89, 95%CI: 0.57, 1.40)”.

**Authors’ response:** We appreciate your attention to accuracy and have revised the statement accordingly:

*“Overcrowded living conditions, malnutrition, shared sleeping arrangements and sharing of clothes and towels with those who are already infected are common risk factors for this disease.” (Page 3, Line 57-59)*

Line 69: Remove “limited access to proper hygiene facilities”. Whilst your reference 6 [Cox et al 2020. B J Dermatol) does discuss communal settings, so is appropriate for this section, it does not mention “limited access to proper hygiene”.

***Authors’ response:*** *Thank you for your careful review. We have removed the phrase “limited access to proper hygiene facilities” from line 69, as the cited reference does not support this and revised the section as:*

*“Communal settings and closed residential environments, like orphanages, where a large number of children live together in the same building or compound, are highly vulnerable for scabies due to factors like crowded living conditions and close contacts with infected peers.” (Page 3, Line 77)*

Line 184: “A recent study conducted among children from islamic residential religious schools reported that almost one third of the children were infected by scabies in these institutions, comparable to our findings [19]. The difference in living condition and hygiene practice among children from different institutions might cause the difference.” Firstly, if you are going to mention this study, for transparency I would like you to insert as follows (capitalisation just to emphasise my addition): “A recent study conducted BY THE SENIOR AUTHOR OF THIS PAPER AND OTHERS among children…” Secondly, remove “and hygiene practice”. As outlined above, this is not supported by the overall evidence base, and infact the study reported in your reference 19 (by one of your own authors) also did not to find any statistically significant association despite between personal washing and scabies, despite looking for it. For example, in Table 3 of Reference 19 its states: “Irregular bathing (yes vs noR) p=0.555; Use soap for baths (yes vs noR) p=0.260” etc.

***Authors’ response:*** *Thank you for your critical observation. We have removed the mention of "hygiene practice", as the overall evidence base and our cited reference do not support this association. revised the sentence for transparency and accuracy as follows:*

*"A recent study conducted by the senior author of this paper and others among children from Islamic residential religious schools (madrasahs) reported that almost one-third of the children were infected by scabies in these institutions, comparable to our findings." (Page 13, Line 245-248)*

Line 198-201: “However, majority of these studies were conducted in community or school setting, while our study was conducted in an institutional setting of orphanages where children belonged to a low socioeconomic condition with crowded living and deprived of adequate personal hygiene and healthcare access.” Given what is outlined above, remove “personal hygiene”.

***Authors’ response:*** *Thank you for your suggestion. We have revised the section as follows:*

*"However, most of these studies were conducted in community or school settings, whereas our study was conducted in an institutional orphanage setting. Children in orphanages often come from low socioeconomic backgrounds and live in crowded conditions, which facilitate close contact with infected peers and increase the risk of scabies transmission. Furthermore, as scabies was primarily diagnosed clinically in the majority of the studies, subjective variations in the diagnostic accuracy of data collectors might contribute to differences observed between the present study and previous research." (Page 14, Line 267-273)*

Line 212: “Personal hygiene is one of the major influencing factors of scabies among children. It is evidenced that children who maintain less personal hygiene, and use shared personal stuffs have a higher odd of having scabies [29], though in the present study, we were not able to demonstrate such association of scabies with factors regarding personal hygiene.” I outline above why your reference 29 says the opposite of what you claim it does, so make the following changes so it accurately reflects reference 29, and your own results. Suggested edit (capitalisation just to emphasise my additions): “Personal BODY WASHING is OFTEN PRESUMED BY LAYPEOPLE AND SOME HEALTH WORKERS TO BE A major influencing factor of scabies. HOWEVER, OUR STUDY’S LOGISTIC REGRESSION MODEL FOUND NO ASSOCIATION BETWEEN SCABIES AND BATHING, WHICH IS IN LINE WITH THE BALANCE OF THE WIDER EVIDENCE BASE.[29] INSTEAD OF PERSONAL WASHING, CONNECTIONS BETWEEN POVERTY AND SCABIES LIKELY STEM FROM HOUSING DENSITY AND LIMITED ACCESS TO HEALTHCARE.[29] NOTABLY, IN OUR STUDY THE STRONGEST POSITIVE ASSOCIATION WITH SCABIES WAS NUMBER OF CHILDREN PER ROOM.”

***Authors’ response:*** *Thank you for your valuable feedback. We acknowledge that the broader body of evidence does not establish a direct association between personal hygiene practices and scabies prevalence. However, some studies, such as Gupta et al. (DOI: 10.1111/tmi.14058), have suggested a potential link between specific personal behaviors, including infrequent bathing and lack of soap use, and scabies. To ensure our discussion accurately reflects the current evidence base, we have revised the section in accordance with your recommendations.*

*“Personal behavioral factors, such as bed-sharing, clothing-sharing, infrequent bathing, lack of soap use, etc. have been suggested as risk factors for scabies in some studies. However, our logistic regression analysis did not find a significant association between these practices and scabies, which is in line with the balance of the wider evidence base. Rather than individual behaviors, the link between poverty and scabies is more likely driven by factors such as housing density and limited access to healthcare. Notably, in our study, the strongest positive association with scabies was the number of children per room. As a contagious disease, the spread of scabies is exacerbated among closely resident and interacting peer groups of children, as evidenced in our study. This aspect highlights the importance of understanding not only individual-level factors but also the dynamics of social interactions in the transmission of scabies.” (Page 14, Line 278-288)*

Lines 250-256 (from your conclusion): “Major factors contributing to this prevalence include male gender, overcrowded living conditions, and close contact with infected peers.” The first sentence here is good, and reflects your findings. However, most of the second sentence does not flow from your findings at all (which should be the basis of your conclusion). You write: “Addressing this public health concern necessitates a comprehensive approach involving interventions aimed at enhancing the hygienic behaviors of children, promoting health education initiatives, and strategically minimizing contact with individuals affected by scabies.” Remove reference to hygiene (looked for, but not demonstrated by your study) and health education (you do not report any survey of knowledge of scabies, so should not recommend education as a panacea). Replace with: “Addressing this public health concern necessitates a comprehensive approach involving interventions aimed at REDUCING DENSITY OF CHILDREN PER ROOM, and strategically minimizing contact with individuals affected by scabies WHEN THEY ARE INFECTIVE.” In addition, I would suggest (though do not require) that you add in something emphasizing the need for mass treatments in affected orphanages, as the prevalence you report (31.63%) is too high to eradicate successfully though individual case management (particularly given that patients will be infective for a substantial part of the initial asymptomatic incubation period). For more information on this see: Engelman et al. 2021. A framework for scabies control. PLoS Negl Trop Dis. doi: 10.1371/journal.pntd.0009661. (NOTE: I am not an author of that paper, so am not recommending something by myself!). The final sentence starting “The findings” is fine as is.

***Authors’ response:*** *Thank you for your thoughtful feedback. We have revised the conclusion to ensure it accurately reflects our study findings. Specifically, we have removed references to hygiene and health education, as these were not demonstrated by our study. Instead, we now emphasize the need for interventions such as reducing the density of children per room and potentiality of mass treatment strategy for better control of scabies in the high burden settings.*

*“In conclusion, our study reported a substantial level of prevalence of scabies among orphanage children in Dhaka, Bangladesh. Major factors contributing to this prevalence include male gender, overcrowded living conditions, and close contact with infected peers. Addressing this public health concern necessitates a comprehensive approach that includes interventions aimed at reducing the number of children per room and strategically minimizing contact with individuals affected by scabies when they are infective. Additionally, considering the high prevalence of scabies in the orphanages, a mass treatment program might better control the transmission of the disease. Finally, the findings of our study would guide the implementation of targeted strategies to mitigate scabies transmission within these settings, thus fostering a healthier environment for the vulnerable population of children in orphanages.” (Page 15, Line 320-359)*

*Besides, we also mentioned the importance of mass treatment strategy in the discussion section as well.*

*“Given the high prevalence of scabies reported in our study (31.6%), relying solely on individual case management is unlikely to achieve effective disease elimination in these settings, particularly where close contact with infected individuals is frequent. Therefore, a more effective strategy such as mass drug administration (MDA) using oral ivermectin for all residents and caregivers might be considered in high-burden settings.” (Page 14, Line 289-301)*

**RE DUPLICATION OF CONTENTS**
Two paragraphs in your discussion section seem to be mainly two different drafts of the same paragraph, covering the same topics and using the same references at the same points, but worded slightly differently. I suspect this is just a simple copy-paste editing mistake. Retain the paragraph on lines 204-215 (making the changes outlined above re hygiene etc.), and delete the following lines in the subsequent paragraph: 216-225.
Then, on line 226 delete “association with hygiene practices. Moreover, being”, and instead start the sentence as follows (capitalisation to indicate my insertions only): “AS a contagious disease, THE spread of scabies is exacerbated among CLOSELY RESIDENT AND INTERACTING peer groups of children, AS evidenced in our study. This aspect highlights the importance of understanding not only individual-level factors but also the dynamics of social interactions in the transmission of scabies.”

***Authors’ response:*** *Thank you for identifying this duplication. We have removed the redundant paragraph. Additionally, we have modified the sentence on line 284 to accurately reflect the role of close residential interactions in scabies transmission, as per your recommendation.*

***I personally feel your study is a useful addition to knowledge of a medically neglected problem affecting the highly disadvantaged; hence my support for your paper but also my attention to its details when you misrepresent that which you are citing, or make recommendations not based on your study findings. I look forward to reading your revised manuscript.***

***Authors’ response:*** *Thank you for your thoughtful and constructive feedback for the improvement of our manuscript. We sincerely appreciate your support for our study and your keen attention to detail, which has greatly helped to improve the accuracy and clarity of our manuscript. We have carefully addressed your comments to ensure that our findings and recommendations align with both our study results and the broader evidence base. We truly value your engagement in strengthening research on this neglected disease and will be happy to further revise the manuscript to ensure its accuracy and rigor.*

***We are open to edit further revision if deemed necessary to improvise the manuscript and made the evidence more concrete.***

**Reviewers' Comments:**

Reviewer's Responses to Questions

**Key Review Criteria Required for Acceptance?**

As you describe the new analyses required for acceptance, please consider the following:

**Methods**

-Are the objectives of the study clearly articulated with a clear testable hypothesis stated?

-Is the study design appropriate to address the stated objectives?

-Is the population clearly described and appropriate for the hypothesis being tested?

-Is the sample size sufficient to ensure adequate power to address the hypothesis being tested?

-Were correct statistical analysis used to support conclusions?

-Are there concerns about ethical or regulatory requirements being met?

**Reviewer #1:**

The sample size was based on estimating the proportion of children with scabies with a gievn precision, which is appropriate. It should be noted that the modelling analyses are exploratory, as the study samples size was not based on the modellling.

Univariable testing should be removed as it is redundant thanks to the regression modelling (see results comments).

There is no reason to arbitrarily set a threshold for "statistical significance". This phrase should not be used, and p-values should be interpreted as strength of evidence against the relevant null hypothesis, rather than dichotomised. This doesn't seem to have been done at all in the paper anyway, so "A value of<0.05 using a 95% confidence interval in logistic regression model was defined as statistically significant level." can be removed from the methods.

***Authors’ response:*** *Thank you for your valuable feedback. In response to your suggestions, we have removed the univariable testing results. Additionally, we have revised the statistical analysis section to avoid arbitrary thresholds for statistical significance and have removed the statement defining p-values dichotomously.*

*“A multiple logistic regression analysis was conducted to identify factors associated with scabies in orphaned children, with odds ratios (ORs) and 95% confidence intervals (CIs) reported.” (Page 7, Line 148-150)*

Need details on data collection - paper CRFs -> databse? What database software?

***Authors’ response:*** *Thank you for your suggestion. We have provided additional details on the data collection process, including how data from paper case report forms (CRFs) were transferred into the database and the specific database software used into the methods section.*

*“Following data collection using a paper-based case record form (CRF), all data were anonymously entered into Microsoft Excel 365 for further analysis. (Page 7, Line 145-146)*

**Reviewer #2:**

The objectives of the study are clearly articulated with a clear testable hypothesis stated. The study design is appropriate to address the stated objectives and the population is clearly described and appropriate for the hypothesis being tested.

The sample size was sufficient to ensure adequate power to address the hypothesis being tested and correct statistical analysis was used to support conclusions. There are no concerns regarding ethical or regulatory requirements.

***Authors’ response:*** *Thank you for your positive feedback. We appreciate your thorough review of our study's objectives, design, and methodology. Your insights are valuable, and we are grateful for your support.*

**Reviewer #3:**

Yes. The study design is suitable for describing the prevalence and the risk factors of scabies in orphanages in the Dhaka city of Bangladesh. It included 471 children from 5 orphanages, which was determined through a sample size estimation. They used the latest criteria of the International Alliance for the Control Scabies, but they made it feasible for its use on the field by only selecting the clinical and suspected scabies criteria. This also applies for the reasoning of not examining some sensible body parts, which then was explained and supported with evidence.

***Authors’ response:*** *Thank you for your thoughtful evaluation of our study design, sample size estimation, and the application of the International Alliance for the Control of Scabies criteria in a field-appropriate manner. Your feedback is greatly valued.*

**Results**

-Does the analysis presented match the analysis plan?

-Are the results clearly and completely presented?

-Are the figures (Tables, Images) of sufficient quality for clarity?

**Reviewer #1:**

Results
Baseline characteristics and Table 1

Mean and SD have been used to summarise all continuous variables, but it’s unclear if these are appropriate, as a number of the variables are counts. Counts are often not normally distributed, so median and interquartile range may be more appropriate.
I think there would be value to showing distribution graphs for these characteristics, so the reader can see the distributions for themselves.
The second heading for table 1 is “n (%)”, but many of the quantities given are mean (SD) – the heading should be changed to reflect this. Add % to any percentage, this will make it obvious when summaries are proportions. Where means or percentages are given with decimal places, just show 1, 2 is unnecessary (same in other tables, but give p-values to 3 d.p.).

***Authors’ response:*** *Thank you for your valuable suggestions. Accordingly, we now report median (IQR) for count variables in the revised tables. Additionally, we have updated the table headings to reflect the appropriate summary statistics and have included mean (SD) and median (IQR) labels for clarity. We have also mentioned the summary statistics beside the name of the variables. We have also standardized the decimal places, using one decimal point for means and percentages while maintaining three decimal places for p-values.*

Factors associated with scabies
The t-tests and chi-square tests are redundant as the results from the regression model are superior. It’s also unclear if the assumptions are met for all of these tests (particularly the normality assumption for t-tests and minimum of 5 expected events (scabies cases) per cell for chi-square). P-values should be removed from Table 3, just summaries and 95% CIs are sufficient.

***Authors’ response:*** *Thank you for your suggestion. We have removed the t-tests and chi-square tests, as the regression model provides a more appropriate analysis. Additionally, we have merged Table 3 and Table 4, retaining only the summary statistics, aORs and 95% confidence intervals, as recommended.*

Logistic regression model
11 variables are included in the model with 149 cases of scabies, which is appropriate. Has collinearity of these variables been checked though? Several of these variables sound like they could be colinear. To check this in Stata you can fit a linear regression model with scabies as the outcome and all the predictor variables, followed by the ‘vif’ command (it does not matter that the outcome is binary, this only considers the predictors). You want to see VIFs <10, otherwise you’ll need to play around with the combination of predictors to identify which are causing high VIFs.

I also think that site (orphanage) should be included in the model to adjust for differences between sites.

***Authors’ response:*** *Thank you for your valuable feedback. We conducted a multicollinearity assessment using the variance inflation factor (VIF) prior to interpreting the multiple logistic regression model. None of the included variables exhibited high collinearity (VIF > 5). However, some variables, such as the number of children in the orphanage, use of the same toilet, and sleeping place, showed moderate VIF values (VIF > 4), suggesting potential collinearity concerns. Given their theoretical significance and relevance to scabies transmission, we retained these variables in the final model.*

*Additionally, we initially included site (orphanage) as a factor to adjust for potential differences across study locations. However, site did not significantly influence the model estimates, and its effect was negligible. Therefore, we excluded it from the final model.*

*These are mentioned in the present version of the Statistical analysis section. (Page 7, Line 148-157)*

**Reviewer #2:** The analysis presented matched the analysis plan and the results are clearly and completely presented. The figures (Tables, Images) have sufficient quality for clarity.

***Authors’ response:*** *Thank you for your feedback. We appreciate your positive assessment of our analysis and presentation.*

**Reviewer #3:**

Yes, the results are clearly and completely presented. They allowed the identification of the prevalence and of the risk factors of scabies in the orphanages in the Dhaka city of Bangladesh. The prevalence was similar as the one reported in other studies, including one that was made in the same city, but in another environment: <https://pubmed.ncbi.nlm.nih.gov/38942588/> The identification of the risk factors allows to develop tailored interventions, which is crucial to tackle its burden. The manuscript only has 1 figure and several tables, but they serve its purpose.

***Authors’ response:*** *Thank you for your thoughtful review and for highlighting the relevance of our findings. We appreciate your acknowledgment of the study's contribution to identifying risk factors and informing tailored interventions for scabies in orphanages. Additionally, we have ensured that the tables and figures effectively present our results.*

**Conclusions**

-Are the conclusions supported by the data presented?

-Are the limitations of analysis clearly described?

-Do the authors discuss how these data can be helpful to advance our understanding of the topic under study?

-Is public health relevance addressed?

**Reviewer #1:**

The conclusions seem generally appropriate.

Some of the predictors do need explicit definitions though -
Iron clothes and bedsheets - how often?
Pruritus in the close entourage - what defines "close entourage"?

***Authors response:*** *Thank you for your feedback. We have now defined the predictors like . "ironing clothes and bedsheets" as ironing at least once a month and "close contacts" as individuals with whom the child had frequent interactions, including sharing a bed for sleeping, exchanging personal belongings, and engaging in academic and recreational activities together. These definitions have been incorporated into the footnote of Table 1.*

**Reviewer #2:**

The conclusions are supported by the data presented. The limitations of analysis and study are clearly described.
The authors discuss how these data can be helpful to advance our understanding of the topic under study and the public health relevance.

***Authors’ response:*** *Thank you for your positive feedback and your appreciation of the study's contributions to advancing understanding of scabies in orphanages and its public health significance.*

**Reviewer #3:**

The conclusions are supported by the data presented and they propose interventions to stop the chain of transmission. Orphanages are environments in which children are greatly exposed to different diseases. It is important to build specific strategies to approach and manage each of them. This study does that. The authors understood the problem and they conducted it in an operative way. They clearly describe its limitations and they do not pretend to make them generalizable for other cities and countries. Local research is valuable. It has public health implications.

***Authors’ response:*** *We appreciate your thoughtful feedback and recognition of the study’s contributions. Addressing transmission of scabies in orphanage settings requires targeted strategies, and we hope our findings provide useful insights for public health interventions.*

**Editorial and Data Presentation Modifications?**

Use this section for editorial suggestions as well as relatively minor modifications of existing data that would enhance clarity. If the only modifications needed are minor and/or editorial, you may wish to recommend “Minor Revision” or “Accept”.

**Reviewer #1:**

The English in the paper is generally good, but there are some odd phrases in some places, such as “personal staffs”, “toilet stuffs”.

“sharing room with a greater number of children” – unclear what this means

“Fingernail always cut short” -> “Fingernails always cut short”

***Authors’ response:*** *We appreciate your careful review of the manuscript and your suggestions for improving the clarity of our language. We have replaced “personal staffs” and “toilet stuffs” with “personal items” and “toiletry items”, respectively. Additionally, we have revised the phrase “sharing room with a greater number of children” as “increased number of children per room”. Furthermore, we have corrected “Fingernail always cut short” to “Fingernails always cut short” for grammatical accuracy. We have carefully reviewed the manuscript for similar linguistic inconsistencies and refined the language to enhance overall readability and clarity. Thank you for your valuable input.*

**Reviewer #2:**

This is a good manuscript with interesting and relevant findings of a neglected tropical disease, affecting most people in low socioeconomic settings of the world.

There is need to make some corrections/amendments to the manuscripts as suggested below:

Line 22: consider changing 'condition' to "setting or status or background". The follwoing sentence change 'The objective of the present study was to' to "This study was conducted to".

Line 25: consider moving parts of the first sentence to results section, i.e. '471 children' and 'five orphanages'. Replace the sentence with "This was a cross-sectional study among children in selected orphanages in Dhaka city of Bangladesh."

Line 30: consider starting the section with this sentence: "471 children living in five selected orphanages of Dhaka city of Bangladesh were screened for scabies. Majority..."

Line 36: consider removing 'Almost'

Line 44: consider adding reference to this sentence; you may wish to move the reference in Line 45 to here

Line 62: consider replacing 'brackets' with "settings"

Line 86: consider replacing 'The present one' with "This"; remove 'five' from this sentence - it should be in the results section.

Lines 100-103: consider moving these lines to results section

Line 139: consider replacing 'the present' with "this"

Lines 143 and 161: consider replacing 'staffs' with "stuff"

Line 162: consider replacing 'entourage' with "company"

Lines 163-170: consider correcting the number on the p-value to 2 decimal places except in very small number (significant result ~ <0.001, <0.003)

Line 173: consider replacing 'the present' with "this"

Line 175: consider adding "s" to 'finding'; remove 'almost'; add "of which" after 'affected by scabies,'

Lines 178-181, 232-247: consider adding references to these sentences

***Authors’ response:*** *We appreciate your meticulous review and specific suggestions to improve the clarity and coherence of our manuscript. We have carefully revised the manuscript throughout to enhance clarity, coherence, and precision in line with your recommendations. Your feedback has been invaluable in refining the presentation of our findings and strengthening the overall readability of the paper.*

**Reviewer #3:**

The manuscript is fluent and well-written and it does only require some minor corrections:
. Line 2. "bangladesh" should be written with an upper case letter: "Bangladesh".
- Line 146. "Scabies" should be written in lower case letters: "scabies".
- Line 241. The "International Alliance for the Control of Scabies (IACS) Criteria" has already been mentioned. It should say "IACS Criteria".

**Authors’ response:** Thank you for highlighting these refinements. We have carefully revised the manuscript to ensure consistency and clarity. Your feedback has been instrumental in enhancing the precision and readability of our work, and we truly appreciate your time and effort in reviewing our study.

**Summary and General Comments**

Use this section to provide overall comments, discuss strengths/weaknesses of the study, novelty, significance, general execution and scholarship. You may also include additional comments for the author, including concerns about dual publication, research ethics, or publication ethics. If requesting major revision, please articulate the new experiments that are needed.

**Reviewer #1:**

I have primarily reviewed the statistics - this paper provides a good summary of the population of interest and answers the question it set out to - the prevalence of scabies in this population. The exploratory logistic regression model is credible (but does require examination of collinearity and inclusion of site) and the results are interpreted appropriately.

**Authors’ response:** We appreciate your review and insightful feedback on the statistical analyses. Your observations regarding the robustness of the logistic regression model and colnearity have been carefully considered in the present version of the ‘Statistical analysis’ section. The necessary assessments have been conducted to ensure the validity of our findings. Thank you for your valuable input in strengthening our study.

**Reviewer #2:**

This manuscript describes an important study on a highly prevalent NTD which affects the most vulnerable and low socioeconomic people in remote, limited resource settings. This study has interesting and significant findings which are very relevant to Health authorities and policy makers in most endemic areas facing the same and similar disease (s).

**Authors’ Response:** Thanks for appreciating the significance of our study and its potential implications for health authorities and policymakers. Your thoughtful feedback reinforces the importance of addressing this neglected tropical disease in vulnerable populations. Thank you for your support.

**Reviewer #3:**

I found this study relevant for public health. Addressing public health problems with general health recommendations that have been made elsewhere is not desirable nor suitable. They can be used to build a framework, but local research is required to tailored them. This manuscript is the perfect example of how to do that. I do hope that the study is replicated in other parts of the country and that it help to improve the health of children who live in orphanages. This neglected tropical disease can be controlled.

**Authors’ Response:** We truly appreciate your thoughtful remarks on the importance of local research in shaping effective public health interventions. We hope our study will provide context-specific evidence in addressing neglected tropical diseases like scabies and improving health outcomes for vulnerable populations.
